# Successful repair of iatrogenic esophageal perforation with novel through the scope closure endoclips

**DOI:** 10.1055/a-2590-7691

**Published:** 2025-05-19

**Authors:** Karthic Drishna Perumal, Raj Shah, Erica Park, Georgios I. Papachristou, Somashekar G. Krishna, Jordan Burlen

**Affiliations:** 1 24596 Internal Medicine, Mount Carmel Health System, Grove City, Ohio, United States; 2 12306 Division of Gastroenterology, Hepatology and Nutrition, The Ohio State University Wexner Medical Center, Columbus, Ohio, United States

Esophageal perforations, a rare but life-threatening emergency, have traditionally required surgery for repair. However, endoscopic therapy has emerged as a promising alternative. This case describes the use of novel through-the-scope appositional clips for closing large full-thickness defects and placing esophageal stents for stricture prophylaxis. A 54-year-old woman with a history of dysphagia was found to have a non-obstructing Schatzki ring at the gastroesophageal junction when evaluated at an outpatient endoscopy center. An 18 mm Savary dilator was used without complication, but blood was noted after removal. A 1.5 cm full-thickness perforation in the lower esophagus was identified on repeat endoscopy, and she was subsequently transferred to a tertiary medical center in stable condition for further management. On arrival at the hospital, she received a computed tomography scan revealing a 2.5 × 3.2 cm esophageal perforation with gas dissecting through the mediastinum. Broad-spectrum IV antibiotics, antifungals, and high-dose IV proton pump inhibitors were administered. During esophagogastroduodenoscopy (EGD), a bleeding perforation 28–33 cm from the incisors was visualized and repaired using 10 hemostatic Mantis endoclips (Boston Scientific) ( Fig. 1 , Fig. 2 ). Then gastrografin was injected without any evidence of a leak. Finally, a 23 mm × 10.5 cm fully covered WallFlex stent (Boston Scientific) was placed for stricture prophylaxis and sutured with through-the-scope suturing to prevent stent migration. Approximately 5 months post-perforation, the patient underwent repeat EGD for a thorough examination of the esophageal mucosa, demonstrating healthy scar tissue at the site of the initial defect. In addition, repeat esophagram studies post-operatively up until 8 weeks after perforation continued to demonstrate no evidence of a leak ( Video 1 ). This case illustrates the efficacy of endoscopic therapy for managing full-thickness esophageal perforations using novel appositional through-the-scope clips and esophageal stents for stricture prevention.

**Fig. 1:**
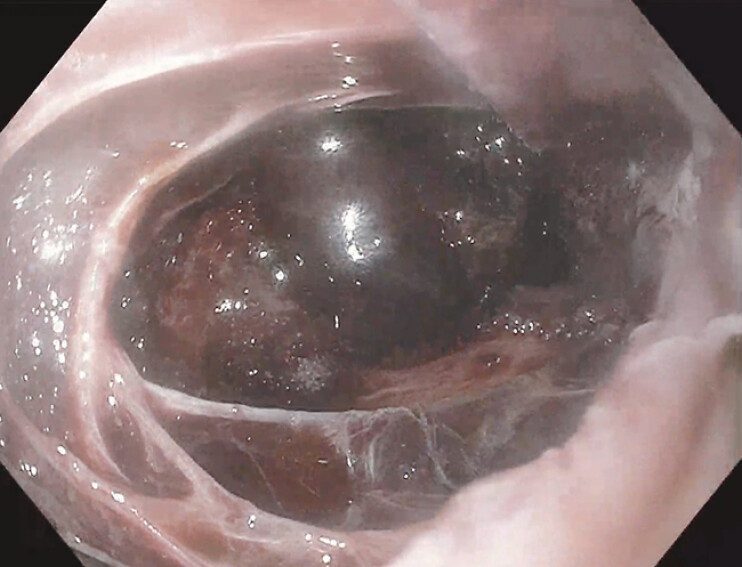
Full-thickness esophageal defect with visualization of the mediastinum.

**Fig. 2:**
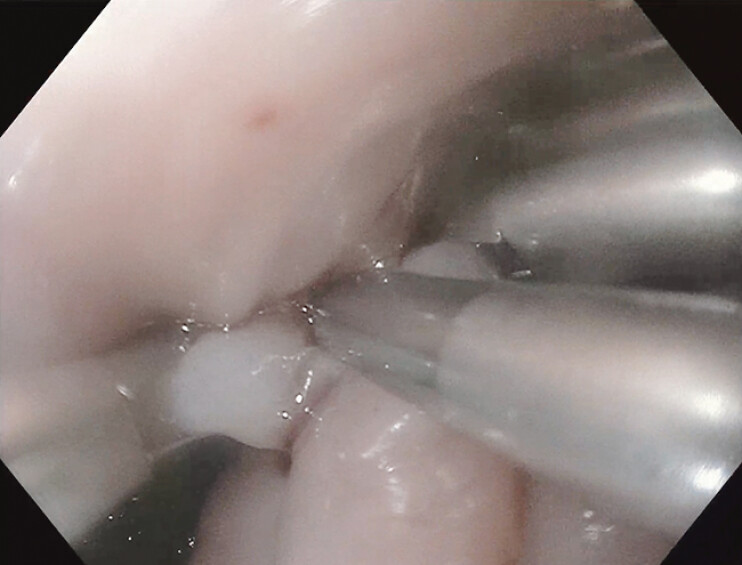
Utilizing Mantis anchor-prong technology to effectively grasp and appose large esophageal tissue segments for successful endoclip application and closure.

Endoscopic repair of full-thickness perforation using 10 Mantis endoclips with subsequent covered stent placement to prevent stricture formation. Repeat EGD and esophagram showing successful tissue approximation and no leak.Video 1

Endoscopy_UCTN_Code_CPL_1AH_2AL

## Notes

Endoscopy E-Videos https://eref.thieme.de/e-videos
*E-Videos* is an open access online section of the journal *Endoscopy* , reporting on interesting cases and new techniques in gastroenterological endoscopy. All papers include a high-quality video and are published with a Creative Commons CC-BY license. Endoscopy E-Videos qualify for HINARI discounts and waivers and eligibility is automatically checked during the submission process. We grant 100% waivers to articles whose corresponding authors are based in Group A countries and 50% waivers to those who are based in Group B countries as classified by Research4Life (see: https://www.research4life.org/access/eligibility/ ). This section has its own submission website at https://mc.manuscriptcentral.com/e-videos .

